# Healthy Indoor Environments: The Need for a Holistic Approach

**DOI:** 10.3390/ijerph15091874

**Published:** 2018-08-30

**Authors:** Aneta Wierzbicka, Eja Pedersen, Roger Persson, Birgitta Nordquist, Kristian Stålne, Chuansi Gao, Lars-Erik Harderup, Jonas Borell, Héctor Caltenco, Barry Ness, Emilie Stroh, Yujing Li, Mats Dahlblom, Karin Lundgren-Kownacki, Christina Isaxon, Anders Gudmundsson, Pawel Wargocki

**Affiliations:** 1 Ergonomics and Aerosol Technology, Lund University, P.O. Box 118, 221 00 Lund, Sweden; chuansi.gao@design.lth.se (C.G.); jonas.borell@design.lth.se (J.B.); karin.lundgren_kownacki@design.lth.se (K.L.-K.); christina.isaxon@design.lth.se (C.I.); anders.gudmundsson@design.lth.se (A.G.); 2 Environmental Psychology, Department of Architecture and Built Environment, Lund University, P.O. Box 118, 221 00 Lund, Sweden; eja.pedersen@arkitektur.lth.se; 3 Department of Psychology, Lund University, P.O. Box 213, 221 00 Lund, Sweden; roger.persson@psy.lu.se; 4 Building Services, Lund University, P.O. Box 118, 221 00 Lund, Sweden; birgitta.nordquist@hvac.lth.se (B.N.); mats.dahlblom@hvac.lth.se (M.D.); 5 Materials Science and Applied Mathematics, Malmö University, SE-205 06 Malmö, Sweden; kristian.stalne@mau.se (K.S.); yujing.li@mau.se (Y.L.); 6 Building Physics, Lund University, P.O. Box 118, 221 00 Lund, Sweden; lars-erik.harderup@byggtek.lth.se; 7 Certec, Lund University, P.O. Box 118, 221 00 Lund, Sweden; hector.caltenco@certec.lth.se; 8 Centre for Sustainability Studies (LUCSUS), Lund University, P.O. Box 170, 22 100 Lund, Sweden; barry.ness@lucsus.lu.se; 9 Occupational and Environmental Medicine, Lund University, Scheelevägen 2, 22 363 Lund, Sweden; emilie.stroh@med.lu.se; 10 Centre for Indoor Environment and Energy (CIEE), Danish University of Technology, 2800 Kongens Lyngby, Denmark; paw@byg.dtu.dk

**Keywords:** healthy indoor environment, holistic approach, transdisciplinary studies, multidisciplinary studies, indoor environment quality

## Abstract

Indoor environments have a large impact on health and well-being, so it is important to understand what makes them healthy and sustainable. There is substantial knowledge on individual factors and their effects, though understanding how factors interact and what role occupants play in these interactions (both causative and receptive) is lacking. We aimed to: (i) explore interactions between factors and potential risks if these are not considered from holistic perspective; and (ii) identify components needed to advance research on indoor environments. The paper is based on collaboration between researchers from disciplines covering technical, behavioural, and medical perspectives. Outcomes were identified through literature reviews, discussions and workshops with invited experts and representatives from various stakeholder groups. Four themes emerged and were discussed with an emphasis on occupant health: (a) the bio-psycho-social aspects of health; (b) interaction between occupants, buildings and indoor environment; (c) climate change and its impact on indoor environment quality, thermal comfort and health; and (d) energy efficiency measures and indoor environment. To advance the relevant research, the indoor environment must be considered a dynamic and complex system with multiple interactions. This calls for a transdisciplinary and holistic approach and effective collaboration with various stakeholders.

## 1. Introduction

The last century’s social, demographic, technical, and political developments have profoundly altered the living conditions of many human populations as well as the patterns of health and disease. In the global north, modern humans spend about 90% of their time indoors [1,2]. Obviously, this has consequences for our daily exchanges with the social and physical environments. While living in closed environments protects us from many of nature’s unwanted fluctuations and exposures, it is also obvious that our artificial environments contribute new risks and create new preconditions that affect human health in new ways. It is therefore important to understand how to design healthy and sustainable indoor environments. Historically, there has been a tendency to explore the effects of one factor at a time, though real-life scenarios are considerably more complex. When focusing on only a single or limited factors, our desired outcome such as energy saving may lead to decreased ventilation and increase in levels of moisture and airborne pollutants indoors, which in turn are likely to cause negative health effects. Thus, having occupants’ health, well-being, comfort, and productivity in mind entails paying attention to a multitude of factors and to a wide range of possible interactions between them and occupant behaviours. Motivated by the absence of previous holistic research perspectives, intending to scrutinize the complexity of the interactions occurring in indoor environments, a multidisciplinary team of researchers collaborated in the project called “Healthy Indoor Environments”. These researchers initially met regularly at the Pufendorf Institute at Lund University in Sweden; they then formed the Centre for Healthy Indoor Environments (www.chie.lth.se). Here they explored the indoor problems through discussions and workshops with invited experts and industrial representatives, which were followed by nonsystematic literature reviews of published peer-reviewed articles within identified areas of interest. Specifically, the project strove to improve the knowledge of indoor environments by applying a holistic perspective to the indoor environment and viewing it as a complex system. 

These researchers contributed with expert knowledge within disciplines such as engineering, medicine, psychology, the thermal environment, environmental psychology, human behaviour, sustainability, the built environment, building physics, material sciences, building services, energy, ergonomics, system modelling, aerosol science, interaction design, universal design, and acoustics. With the objective to allow unprejudiced and innovative thoughts, no specific method or formally defined procedure was followed in the project and the themes discussed emerged primarily as a consequence of the represented expertise, input from various stakeholders, available research methods and the complex problem area as such. While gathering many disciplines to tackle indoor-related problems has great appeal, it is not a frictionless endeavour. In our initial multidisciplinary approach, one difficulty was to manage the trade-off between the precision and breadth of the definitions and concepts used [3]. Finding precise definitions on which researchers from different disciplines can agree can be challenging. However, as Van de Ven [4] pointed out, demanding excessive exactness can prematurely limit the development of ideas/concepts when theorizing. In the early stages of multidisciplinary work, one should be somewhat relaxed regarding the formulation of exact definitions that suit everyone. In fact, accepting broad concepts may actually make it easier to build a theory or model that takes thinking to the next step. For example, terms such as “indoor air quality” and “health” can be used in a simplified or more open-ended manner, although their definitions can still be questioned and discussed. To approach such a vast and complex subject area, certain simplifications are necessary in order to gain an overview and create a framework. Some details can be refined later in the process as needed, whereas other details may never be addressed perfectly. Other common method such as a systematic literature review with predefined keywords was not deemed as the most beneficial in our project, we wanted to avoid setting any boundaries or limits in order to allow new important aspects to emerge. 

Rather than promoting a healthy indoor environment by suggesting quick fixes or applying established research methods that give precise results within the boundaries of any limited research field, our aim is to open up a broader discussion and present a change of perspective in evaluating indoor environment quality. We present therefore several perspectives supported by literature from various fields and demonstrate how they may interact. In this paper we summarize some central outcomes of our project and highlight components that in our opinion are needed in further research to ensure a holistic approach to indoor environment issues. Four themes of special interest emerged and were discussed from the perspective of interaction between factors and the potential risks of not considering them holistically: (a) bio–psycho–social aspects of health; (b) interaction between occupants, buildings, and indoor environment quality; (c) climate change and its impact on indoor environment quality, thermal comfort, and health; and (d) energy-efficiency measures and design to achieve good indoor environment quality. Below we discuss each theme in turn, advancing from theoretical models and coping behaviours, through occupant interactions with building systems, to specific examples of interaction between various factors and their potential consequences for indoor environment quality and human health. An intended overall aim of the paper, as well as for the Centre for Healthy Indoor Environments, is to facilitate an increased awareness of the complex nature of the indoor environmental problems and demonstrate the virtues of a holistic approach, even for researchers that intend to stay within the respective boundary.

## 2. Themes of Special Interest Discussed from the Perspective of Interaction between Factors and the Potential Risks of Not Considering Them Holistically

### 2.1. Bio–Psycho–Social Aspects of Health

A central rationale for research into the indoor environment is to prevent discomfort and increased health risk for the occupants. Unsurprisingly, health was a recurring discussion point throughout our eight-month project. To understand our discussion of health, general developmental trends in population health need to be considered. Indeed, in industrialized countries, the population is expected to live longer, dying mainly from non-communicable chronic diseases, such as cardiovascular diseases, cancer, diabetes, and chronic lung diseases [5]. Some of the observed shifts in health and disease patterns are attributable to better buildings and indoor conditions, yet many other factors have acted in concert over time [6]. Aside from improved sanitation and housing, such factors include better health services, better diagnostic tools and medical treatment regimens, better nutrition, changed sleep, work, and activity patterns, and the introduction of novel chemical substances. 

However, not only has public health improved, but recent developments have necessitated a broader view of health that considers psychological and social aspects and views health as an illness–wellness continuum rather than a dichotomy (i.e., healthy vs. sick) [7]. A leading proponent of this expanded view of health was Engel [8], who suggested the bio–psycho–social (BPS) model. On one hand the BPS model is about understanding how suffering disease and illness may depend on multiple levels of organization. Poor health can therefore be viewed as resulting from the complex interaction of diverse causal factors at different levels, including the molecular, individual, and social levels [8,9]. On the other hand, the BPS-model is also about understanding the individual’s subjective experience as a contributor to accurate diagnosis, health outcomes and human care. As such, the model emphasizes that psychosocial variables are important determinants of the susceptibility to, severity of, and course of illness [9]. People are thus active agents and their introspective accounts should be taken seriously, even if their subjective experiences do not correspond to detectable changes in physiology. Accordingly, there are good reasons to consider introspectively assessed correlates of health (e.g., comfort, well-being, self-esteem, anxiety, depression, and other forms of symptom reporting) and to seek associated expressions at different organizational levels (e.g., the individual, group, organizational, and societal levels).

A fundamental concept in understanding health is “*physiological balance*”, sometimes denoted homeostasis or allostasis [10,11,12,13]. According to Nelson [14], the two most important factors in maintaining a reasonable physiological balance over the life-course are proper nutrition and sleep. The physiological balance may be compromised both by direct effects on physiology (e.g., not receiving food or water) and by psychological stimuli such as expectations and beliefs (e.g., worrying before an event). 

Achieving physiological balance may be obtained by activating physiological systems (e.g., the hypothalamic–pituitary–adrenal axis) or by behavioural efforts (e.g., eating, sleeping, drinking, or wearing extra clothes or opening the window when the indoor climate is perceived as too cold or warm) [14]. A complicating factor is that the list of potential events and circumstances that may perturb the physiological balance is in principle infinite. For example, the physiological balance may be disturbed by environmental exposures to sound, airborne particles, and odour, by diseases, obesity, poor social relations, and perceived job insecurity. In addition, not all imbalances can be considered harmful, as they may merely reflect natural adaptive responses and thus represent normal short-term reversible adaptations. Indeed, biochemical alterations do not directly translate into disease [9]. However, if the perturbations that cause physiological imbalances are too frequent and prolonged, poor health may well result as the necessary psycho–physiological restoration is hindered. 

When it comes to understanding the health–indoor environment relationship, much previous research has focused mainly on acute health symptoms and discomfort, and to some extent on cognitive performance. Part of the associated knowledge is reflected in current building standards related to the indoor environment. Yet there are large knowledge gaps and many unanswered questions concerning how to define specific features of “healthy buildings” and how to understand the complex interactions between indoor environments, health, energy efficiency, and sustainability. For example, defining, understanding, and interpreting what constitutes a healthy building depend partly on which actor (e.g., occupants, building contractors, architects, and health and safety inspectors) is evaluating the situation. Different actors are likely to have different perspectives and motives and thus different criteria for defining a healthy indoor environment. To capture these different perspectives when creating indoor environments, understanding the bio–psycho–social aspects of health is a prerequisite. 

### 2.2. Interaction between Occupants, Buildings, and the Indoor Environment, and How These Are Affected by Occupant Understandings of Building System and Functionality

#### 2.2.1. Human Appraisal of the Environment and Subsequent Coping Behaviour

It is clear from the previous section that the indoor environment should provide opportunities to maintain or regain psychological and physiological balance. In addition to the purely physical dimensions of the environment, occupants and their behaviour influence the indoor environment, which in turn can have both positive and negative effects on health. Human behaviour relative to the indoor environment can be understood as constant interaction between the physical environment and the individual [15]. According to cognitive stress theory as proposed by Lazarus and Folkman [16], individuals appraise the qualities of the existing indoor climate (e.g., noise, air quality, lighting, and temperature) as beneficial, neutral, or threatening. If the indoor environment is perceived as threatening, a secondary appraisal generates an inventory of available coping strategies, which in turn may result in active coping behaviours (e.g., turning off ventilation to decrease noise) or in resignation [17]. Resignation or perceiving the environment as incongruent with one’s needs and wants can trigger or prolong adverse stress reactions that negatively affect quality of life [18]. Active behaviour will decrease the psychological stress, but if wrongly targeted may also increase the risk of adverse health effects by creating unwanted or unexpected side-effects (e.g., lower air quality). While cognitive interpretations and appraisals are often thought of as being rapidly made without any special awareness, they build on knowledge of the environment and on personal goals and beliefs. Knowledge of health risks, personal motives (e.g., caring about the health of one’s family), and perceived opportunities (e.g., other ways to control noise) are significant prerequisites for desired behaviour [19]. 

#### 2.2.2. Examples of Interaction between the Indoor Environment and Occupants

Insufficient air exchange rates are sometimes found in housing equipped with mechanical ventilation [20,21], not just because of technical deficiencies, such as unbalanced ventilation systems, but also from human interaction with the systems [22]. In previous studies, residents reported having insufficient interest or knowledge to control ventilation systems, so they did not increase the air exchange rates when needed [22]. Residents have also reportedly found noise from ventilation systems so irritating that they reduced the ventilation rates to eliminate the noise [23]. One possible consequence is that the occupant may perceive the indoor air as stuffy and adjust the conditions by, for example, opening a window [24], a common action even in the winter in a country, such as Sweden, with a cold climate [22]. Not only does this increase energy use, it also, in urban areas, increases the amount of outdoor air pollutants infiltrating indoors [25]. Active coping behaviour, such as reducing noise exposure by reducing the ventilation rate, could lead to a secondary adverse exposure, such as insufficient air quality, evoking yet another coping behaviour, such as opening a window, resulting in higher levels of outdoor pollutants infiltrating indoors. 

#### 2.2.3. The Interface between Physical Parameters and Humans

The interface between physical parameters and occupants is crucial allowing the occupants to exert control over the indoor environment as described in the examples mentioned in Section 2.2.2. When designing for healthy indoor environments, the link between the physical parameters of the indoor climate and humans can be seen as an interface, bi-directionally connecting humans to their physical surroundings. Fundamental to such an interface is that it should be understandable to users and that the users should feel in control of the system [26,27,28]. Adequate feedback to users is considered an important element of environmental control systems [29]. Delayed or incorrect feedback impedes the users from understanding and controlling the systems [30], while adequate feedback can lead to better utilization of the system, energy savings and reduced emissions [31]. In this context, it is important to emphasize that though permissible exposure levels exist for certain emissions, there is a large research gap concerning the human health effects of many other emissions, especially when they occur together or in combination with other factors. 

However, it is important to acknowledge that the occupants of indoor environments are not homogenous. Extending the above example, air-related practices have been described as activities that, besides having purely functional rationales, also touch on prominent personal and social values [32]. The reasons for opening windows may be as diverse as to connect to nature, hear birds or the “urban pulse”, manage odours, or feel a breeze [33]. Individual variation is thus yet another parameter to include in a holistic perspective. 

The ongoing development of “smart homes” incorporates the use of sensors and advanced control systems to adapt various functions and indoor climate parameters in dwellings to their occupants’ varying needs and wishes [34]. To provide the occupants with a healthy indoor environment, such attempts require not only advanced technical equipment [35,36] but also knowledge of the occupants’ preferences and behavioural habits. This knowledge could be attained by applying principles that acknowledge the diversity of people’s needs and preferences [37]. The systems must be intuitive and understandable to occupants, and must be created based on both interactive and universal design principles. It may be that different approaches, by means of specific guidelines, hands-on training, and schemes to educate occupants, need to be considered, as the general understanding of the importance of indoor environment quality and its influence on health, comfort, and productivity is generally poor. 

### 2.3. Climate Change and Its Impact on Indoor Environment Quality, Thermal Comfort and Health 

#### 2.3.1. Challenges in the Context of Climate Change

The previous sections focused on the indoor climate as part of the building system, and acknowledged the role of the occupants, but a holistic view also embraces global environmental factors. Ongoing climate change is predicted to lead to more frequent and severe heat waves and to increased precipitation in some parts of the world [38]. This will affect buildings and indoor environments, leading to direct and indirect consequences for occupant health, comfort, and performance. Direct exposure in the form of heat stress has well-documented adverse health effects and is responsible for excess mortality among exposed persons [39,40,41,42]. Higher temperatures will indirectly affect outdoor air pollution levels by means of increased emissions, for example, of pollen, and increased chemical reaction rates leading to the formation of secondary pollutants [43,44]. Occupants of buildings without air conditioning and effective filters may experience heat during summer, as well as higher levels of outdoor air pollution infiltrating indoors when window opening is used to mitigate the consequences of elevated temperatures. Exposure to outdoor air pollution has various negative health effects; notably, outdoor air pollution has been classified as a human carcinogen [45]. Additionally, higher indoor temperatures will increase emissions of organic substances from building materials, resulting in higher concentrations of gas-phase pollutants that can react with existing gases and particles indoors and that are available for subsequent oxidation reaction chains that form secondary pollutants in both the particle and gas phases. Due to higher temperatures, both the amount of chemicals available for reactions in indoor air and the rate of these reactions will increase. Such exposures in indoor environments, strongly influenced by indoor air chemistry, have received increased attention as there are concerns regarding their health effects [46,47]. 

The described effects of climate change will not affect all people equally. The elderly, children, chronically ill [48,49], pregnant women [50], people with disabilities or on medication [51], the economically disadvantaged, workers with heavy physical loads or protective clothing [52] and city dwellers are much more vulnerable both to exposure to temperature extremes and to the effects of such exposure than are the other population groups [41]. For example, epidemiological studies in elderly population in Sweden and USA show that elevated ambient temperatures and heat waves are directly associated with increased mortality in this group [53,54,55].

#### 2.3.2. Air Temperature and Thermal Comfort as a Complex System

Indoor temperatures follow outdoor temperature changes particularly in free-running buildings [42,56,57]. However, temperatures indoors can vary significantly relative to temperatures outdoors, being up to 50% higher than those outdoors [58,59]. Literature reviews indicate that the relationship between outdoor temperatures and indoor temperatures in non-air-conditioned buildings is complex, depending on interaction between natural processes and technical systems, building design, and passive cooling measures such as solar shading, individual behaviour, and social systems [44,60,61]. For the occupants of the building, thermal comfort is very important and is often ranked as more important than visual and acoustic comfort and good air quality [62]. Thermal comfort does not depend on temperature only. Body heat balance and thermal comfort are influenced by climate factors (i.e., air temperature, humidity, air velocity and radiation), personal factors such as intensity of physical activity (i.e., metabolic heat production) and clothing insulation [40,63], as well as preferences, experience and past exposures. Human thermal comfort is the condition of mind that expresses satisfaction with the thermal environment [64]; it is not related to a particular temperature set-point or narrow interval, but is determined by multiple factors as described above. Regulating the indoor temperature to a fixed interval might not be optimal from the occupants’ viewpoint or from the perspective of energy consumption. At a constant air temperature, any change of other climate factors will influence the thermal comfort; for example, exposure to solar radiation through large windows during the day and extra heat loss at night result in large temperature swings [65,66,67]. As the primary goal of heating or cooling the indoor environment is to create thermal comfort [68], all factors influencing thermal comfort (as described above) should be taken into account and studied as the complex system they constitute. 

#### 2.3.3. Applied Strategies and the Risk of New Adverse Effects

To cope with increased heat waves resulting from climate change, strategies on the macro scale (e.g., technical and design solutions) or the individual level (e.g., adaptation) can be applied. Macro-scale measures include solar screening, reflecting materials and paints on outdoor surfaces, high available thermal mass, shade trees [69,70], air-conditioning based on solar energy [71,72], shutting off or bypassing heat recovery in the summer, and phase-change materials [73]. These measures seem unproblematic. However, it has also been suggested that ventilation systems not equipped with cooling could be switched off during the daytime to avoid drawing in hot air from outdoors [74]. This can be effective and justified as a short-term measure during extreme heat waves, but if commonly used it can create new adverse health effects [75]. By switching off ventilation during the daytime, pollutants, bioeffluents, and moisture will accumulate and indoor air quality will decrease, negatively affecting occupant comfort and health. Increased moisture levels may also lead to mould growth, which can cause material damage and increase the risk of adverse health effects. Such shortcuts (i.e., using short-term measures as permanent solutions) are unfortunately not uncommon, as decisions on strategic solutions are often made based on specialized but frequently fragmented knowledge lacking a holistic perspective. Combined with a constant drive to minimize the use of economic resources (e.g., personnel and allocated time), this could lead to new adverse effects in the long term as in the example described above. In fact, the measures taken to mitigate the effects of acute exposure, in this case heat, could from a longer-term perspective have even worse effects on human health and in terms of damage to buildings and materials. This example illustrates the complexity of the problem and the need for an integrated and multifaceted approach not focused on addressing just one aspect at a time. 

On an individual level, measures targeting one or more of the factors influencing thermal comfort may alleviate heat stress. Possible solutions include opening or closing windows, creating draughts [76] taking cold showers, installing personal fans [61,75], or using personal cooling devices such as cooling vests [77,78]. These measures can improve thermal comfort and help people cope with heat waves, but the use of certain solutions (e.g., electric fans) individually or in combination should be further studied under real-life conditions to capture their combined effects [79], including effects on occupant productivity, discomfort due to noise or indoor air quality, and changed behaviour. Most of these measures also result in higher electricity consumption, which in many countries is associated with increased fossil-fuel combustion and greenhouse-gas emissions, in turn increasing the risk of heat waves, though this is a significantly slower feedback process. It is crucial always to evaluate suggested strategies to combat one problem, in this case the negative health effects of heat waves, using a reflective holistic approach to indoor environments to avoid unexpected adverse outcomes.

### 2.4. Energy-Efficiency Measures and Indoor Environments

#### 2.4.1. Observed Risks Associated with Energy Efficiency Measures in Buildings

As part of the effort to mitigate climate change, energy-efficiency measures have been introduced in the building sector. This is justified, as much of the energy used in western countries is spent on heating, cooling, and regulating indoor environments in buildings [80]. One problem is that existing buildings often have poorly insulated (i.e., low U-value) building envelopes that allow unwanted air leakage. Energy-efficiency measures therefore include sealing the building envelope, increasing thermal insulation, and changing windows. These measures can be problematic, however. In general, thicker thermal insulation (installed from indoors) means that more of the envelope will be influenced by the high relative humidity outdoors during the cold part of the year in countries with climates similar to that of Sweden, which might increase the risk of mould growth [81]. As a consequence of the greater internal insulation, the original parts of the envelope will be more exposed to the outdoor climate, increasing the risk of moisture problems [27]. In addition, in new construction, the demand for low or zero energy use results in well-insulated and sealed buildings, sometimes using previously untested materials. It is known that extensive thermal insulation in wood-framed walls will lead to longer dry-out times for both built-in moisture and moisture from precipitation and leakage [82]. If the dry-out times are prolonged, the risk of mould and other moisture-related problems will increase. Refurbished or newly built sealed homes with optimized ventilation may lead to lower ventilation rates than intended due to occupant interventions, for example, as described earlier, to reduce noise. Decreased ventilation rates in buildings provide insufficient dilution and removal of pollutants, allowing accumulation of pollutants indoors. Airborne pollutants indoors do not originate from the outdoors only; they are also generated indoors in large amounts due to direct emissions (e.g., from cooking, candles, and cleaning products) and are also formed as secondary pollutants due to chemical reactions occurring indoors [83,84]. Decreased ventilation provides more time for such reactions to form new pollutants and by-products that may be irritating for humans. Indoor pollutant sources and indoor air chemistry are rapidly expanding areas of research, highlighting the need to consider indoor pollution in assessments of exposure and possible health effects, areas that have so far been neglected. Decreased ventilation will not efficiently remove moisture either, which in turn may lead to mould growth, moisture-related health problems, and building material damage.

#### 2.4.2. Problems beyond Physics and Biology

Insulating a building and sealing its envelope, especially in combination with energy-efficiency measures to reduce the air flow rates, could lead to indoor environments that are directly unhealthy. There is currently extensive knowledge, reflected in building regulations, of how to prevent excessive moisture levels, high relative humidity, and condensation and mould growth on solid materials. Despite this, the media continue to report on refurbished and newly constructed buildings that must be evacuated and remediated or, at worst, demolished because of mould problems. It seems as though focusing on one aspect, in this case energy efficiency, displaces attention paid to other aspects, such as preventing mould growth and ensuring healthy indoor air quality. In a workshop held with practitioners as part of our collaboration, it was also clear that construction industry requirements for short delivery times and low costs create a situation in which it is difficult to follow recommendations that include longer drying times. The demand for new, less-energy-consuming cement has resulted in new moisture-related problems [85], again confirming that a measure beneficial according to one parameter could lead to unexpected adverse consequences according to others.

#### 2.4.3. Bridging the Gaps

Clearly, several gaps need to be addressed. Within academia, researchers studying the same objects or phenomena need to transfer basic knowledge and understanding across disciplines to build in-depth understanding and avoid conveying conflicting conclusions and recommendations to stakeholders. Bridging the disciplines, multidisciplinary approaches and integrated strategies have started to emerge in scientific literature [86,87,88,89,90]. Another well-known gap is that between scientific outcomes, on one hand, and societal regulation and praxis, on the other. Academia has a major responsibility to deliver results and conclusions in a form accessible to those in a position to influence people’s indoor environment in various ways (for examples, see www.chie.lth.se). The problem of unrealistic conditions in the construction industry must be addressed on several fronts. 

## 3. Conclusions

### Components Needed to Advance Future Research into Indoor Environments

In this paper, we have briefly summarized parts of our work within the multidisciplinary project “Healthy Indoor Environments”. This project was launched to apply a holistic perspective in indoor environmental research, and to scrutinize the complexity of the interactions occurring in indoor environments. In the selected scenarios, we discussed theoretical models, coping behaviours, environmental factors, and occupant interactions with the indoor environment as a system, focusing on the interactions occurring and risks incurred if these are not considered from a holistic perspective. Although we have striven to apply a holistic approach, this approach should be seen as an ideal and not as completely realized, as several aspects, for example, economic and political factors, have not been considered. As a final comment, we present the arguments for considering components that we believe might help advance research and that should be included in future studies of indoor environments. 

First, to help ensure that everyone has access to safe and healthy buildings, *a holistic approach* to indoor environments is needed. Such an approach should encompass: (i) physical parameters, technical systems, and occupants; (ii) the interaction between these; (iii) the societal context in terms of building regulations and industrial organization; and (iv) global environmental issues. Even studies focusing on one or a limited number of aspects of the indoor environment need a holistic awareness of the problem to ensure that problems are properly demarcated and that assumptions concerning the variables and interactions are considered within a wider context. A further motivation for having a holistic awareness is to avoid measures, some of which are described here, that benefit some aspects of the indoor environment while harming others, possibly resulting in a net decrease in occupant health.

Second, to bridge disciplinary gaps and advance our understanding of how technology systems interact and of how people interact with these systems, there is a need for true *transdisciplinary research*. Mere multidisciplinary collaboration in which multiple researchers study indoor environments from their own perspectives in parallel is not enough. Theories and experiences must be transferred and used as a basis for research work and progress, to ensure that research results are not misinterpreted or incorrect conclusions or solutions proposed, so that knowledge is genuinely improved and a holistic approach is ensured.

Lastly, there is a need to *collaborate with various stakeholders*, such as construction market, municipal, and central government actors—this is the only way to understand existing problems and to foster improved knowledge. Dialogue must take place in a way that is understandable and accessible to all parties. In view of extensive worldwide research initiatives regarding minimizing energy use in buildings, smart homes, smart cities, and climate change adaptation and mitigation, we hope that the aspects highlighted here will be considered in future research.
